# Effect of tDCS Over the Right Inferior Parietal Lobule on Mind-Wandering Propensity

**DOI:** 10.3389/fnhum.2020.00230

**Published:** 2020-06-18

**Authors:** Sean Coulborn, Howard Bowman, R. Chris Miall, Davinia Fernández-Espejo

**Affiliations:** ^1^School of Psychology, University of Birmingham, Birmingham, United Kingdom; ^2^Centre for Human Brain Health, University of Birmingham, Birmingham, United Kingdom

**Keywords:** transcranial direct current stimulation, mind-wandering, default mode network, sustained attention to response task, inferior parietal lobule, task-unrelated thoughts

## Abstract

Mind-wandering is associated with switching our attention to internally directed thoughts and is by definition an intrinsic, self-generated cognitive function. Interestingly, previous research showed that it may be possible to modulate its propensity externally, with transcranial direct current stimulation (tDCS) targeting different regions in the default mode and executive control networks (ECNs). However, these studies used highly heterogeneous montages (targeting the dorsolateral prefrontal cortex (DLPFC), the right inferior parietal lobule (IPL), or both concurrently), often showed contradicting results, and in many cases failed to replicate. Our study aimed to establish whether tDCS of the default mode network (DMN), *via* targeting the right IPL alone, could modulate mind-wandering propensity using a within-subjects double-blind, counterbalanced design. Participants completed sustained attention to response task (SART) interspersed with thought-probes to capture their subjective reports of mind-wandering before and after receiving anodal, cathodal, or sham tDCS over the right IPL (with the reference over the left cheek). We found evidence for the lack of an effect of stimulation on subjective reports of mind-wandering (JZS-BF_01_ = 5.19), as well as on performance on the SART task (errors (JZS-BF_01_ = 6.79) and reaction time (JZS-BF_01_ = 5.94). Overall, we failed to replicate previous reports of successful modulations of mind-wandering propensity with tDCS over the IPL, instead of providing evidence in support of the lack of an effect. This and other recent unsuccessful replications call into question whether it is indeed possible to externally modulate spontaneous or self-generated cognitive processes.

## Introduction

Mind-wandering refers to the diversion of one’s attention away from ongoing task demands towards self-generated or spontaneous internally directed thoughts (Smallwood and Schooler, 2015). We typically spend 25–50% of our waking hours engaged in mind-wandering (Killingsworth and Gilbert, 2010; Song and Wang, 2012), and yet the focus of cognitive neuroscientific research has largely been on goal-oriented and externally directed thoughts (Callard et al., 2013). Over recent years, however, there has been increasing interest in elucidating the neural correlates of mind-wandering and determining whether it is possible to modulate this spontaneous process using external sources, such as transcranial direct current stimulation (tDCS).

Most research suggests that mind-wandering relies upon activation of the default mode network (DMN; Christoff et al., 2009), which includes the medial prefrontal cortex (mPFC), posterior cingulate cortex/precuneus (PCC) and bilateral inferior parietal lobule (IPL; Boly et al., 2009). Nevertheless, there is evidence that mind-wandering can also lead to the activation of the executive control network (ECN; Christoff et al., 2009; Golchert et al., 2017). The ECN is characteristically involved with undertaking cognitive demanding tasks, such as response inhibition tasks (e.g., go/no-go; Christoff et al., 2009), attentional and mental reasoning tasks (e.g., Stroop; Dobrynina et al., 2018) and working memory tasks (e.g., n-back; Mencarelli et al., 2019), all of which see their performance greatly reduced with mind-wandering (Unsworth and McMillan, 2014). While the above studies (Christoff et al., 2009; Golchert et al., 2017) suggest both the DMN and ECN may indeed interplay in the generation of mind-wandering, their individual contributions, and the specific dynamics of the relationship with each other, still remain unclear.

To our knowledge, seven recent studies tackled this question by using tDCS to investigate causal roles of DMN and ECN in mind-wandering (Axelrod et al., 2015, 2018; Kajimura and Nomura, 2015; Kajimura et al., 2016, 2019; Bertossi et al., 2017; Boayue et al., 2020). Mind-wandering was measured *via* experience sampling techniques, whereby participants self-reported their internal state in response to thought-probes (Giambra, 1995; Smallwood and Schooler, 2006) periodically presented during a tedious and monotonous task (Christoff et al., 2009; Kajimura et al., 2016). In two different studies totaling 132 participants across different stimulation conditions, Axelrod et al. (2015, 2018) reported a successful modulation of mind-wandering after tDCS over the left dorsolateral prefrontal cortex (DLFPC). Specifically, they found that anodal tDCS leads to an increase in mind-wandering during sustained attention to response task (SART). The authors attributed this modulation to the ECN’s involvement in mind-wandering. However, a recent multi-center registered report with a large sample (*n* = 192) was unable to replicate Axelrod et al.’s (2015, 2018) findings, and instead provided evidence supporting the lack of an effect of DLPFC-tDCS on mind-wandering propensity (Boayue et al., 2020).

Subsequent research reported successful modulations of mind-wandering after concurrent stimulation of the DLFPC and right IPL (Kajimura et al., 2016). Due to the nature of the montage, which targeted regions in both DMN and ECN, the authors were unable to conclude which network was driving the observed changes. However, in a subsequent study, they reported modulation of mind-wandering during a SART task *via* stimulation over the right IPL only, using a reference electrode over the left buccinator muscle (Kajimura et al., 2019). Specifically, anodal stimulation decreased mind-wandering compared to sham. The authors related this effect to changes in the effective connectivity of the DMN at rest. Although promising, these studies do not offer conclusive evidence for the effect of tDCS over the IPL on mind-wandering due to several limitations. Specifically, they failed to control for polarity, including only anodal and sham conditions. Most importantly, their design does not provide adequate control for individual variability on mind-wandering propensity, in that they only measured mind-wandering after tDCS without considering baseline levels.

To address these limitations, here we aimed to further investigate whether tDCS stimulation of the right IPL can indeed modulate mind-wandering propensity in a 3-session, within-subjects design in which we could compare anodal, cathodal and sham stimulation. We used a SART task interspersed with thought-probes both before and after tDCS. We hypothesized that anodal stimulation would result in a reduction in mind-wandering propensity compared to sham and that the effect would be reversed for cathodal stimulation.

## Materials and Methods

### Participants

Thirty-three participants volunteered to take part in the study from an opportunity sample at the University of Birmingham. Participants provided written consent and received course credits for their participation. Before their inclusion in the study, we screened all participants for tDCS safety. The University of Birmingham’s Science, Technology, Engineering, and Mathematics Ethical Review Committee provided ethical approval for the study.

Four participants failed to complete all sessions and their data was removed from the study. We also removed the data from a further participant after they reported at the end of their first session that they had not understood the task. After performing quality control on the remaining data, we removed five participants who failed to respond for 30 s or more of the go/no-go task, suggesting a lack of engagement and compliance with the task instructions. We analyzed the remaining 23 participants’ data (six males, aged 18–23; *M* = 19.83, SD = 1.34). Our sample size was determined based on previous studies using tDCS over IPL to modulate mind-wandering (Kajimura and Nomura, 2015; Kajimura et al., 2016, 2019). Our number of participants per condition was comparable to previous between-subjects studies (Kajimura and Nomura, 2015; Kajimura et al., 2016) and 2-fold greater than the only previous within-subject study (Kajimura et al., 2019).

### Materials

#### Brain Stimulation

We administered tDCS *via* a NeuroConn DC-Stimulator (neuroCare Group GmbH), using 5 × 5 cm rubber electrodes covered with saline-soaked sponges. We placed one electrode over P4 (according to the standard 10-20 system for electroencephalography electrode placement) and the other over the left cheek, as used in Kajimura et al. (2019; NB. their electrode size was 5 × 7 cm). The position of the electrodes was reversed for the anodal and cathodal conditions (i.e., anodal electrode over P4 or the left cheek, respectively) and counterbalanced for the sham condition (i.e., half of the participants received an anodal montage and the other half received a cathodal montage in a counterbalanced order). Before applying the electrodes, we cleaned the skin with alcohol wipes. To identify the location of P4 on each participant and session, we used a customized EEG cap and marked P4 with a pen on the participant’s scalp. After that, we removed the cap and fixed the electrodes in place using Coban tape.

Participants received 20 min of continuous 1.5 mA stimulation with 10 s fade-in and fade-out periods, as per previous research (Kajimura et al., 2019). We monitored impedance and made sure it stayed below 15 kΩ.

For the sham condition, stimulation was delivered for 30 s before fading out to give the sensation of active stimulation. We used a double-blind design, using the “study mode” feature of the device (i.e., each stimulation session was linked to an arbitrary code which would be entered into the machine and deliver real or sham stimulation that would be blind to the researcher and participant). This is in contrast to Kajimura et al. (2019) where only participant-blinding was used. At the end of each stimulation session, participants completed a post-tDCS perception questionnaire in which they reported their perceived sensations and their impression about whether they had received real stimulation or sham.

#### Electric Field Estimation Using SimNIBS

**Figure 1:**
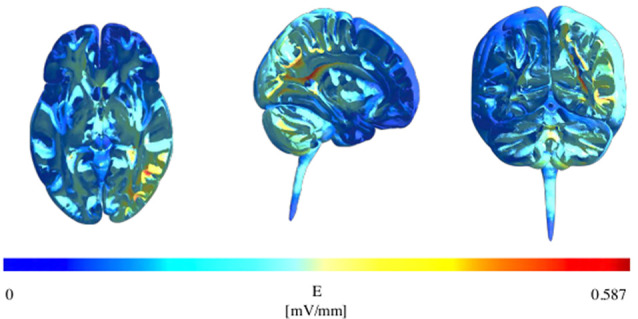
Modelling of electric field strength showing maximum field strength over the right inferior parietal lobule (IPL) in the axial, sagittal, and coronal planes.

We created a finite element method (FEM) head model using SimNIBS v2.1.0^1^ using conductivity values for various tissues as described in Opitz et al. (2015), and modeled on the electrode montage previously described (P4 and left cheek, 1.5 mA). This confirmed that our montage resulted in a peak electric field strength over the right IPL as displayed in Figure 1.

#### Sustained Attention to Response Task (SART) and Thought-Probes

**Figure 2:**
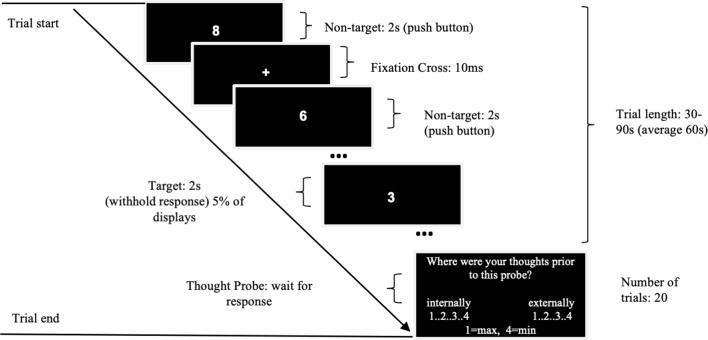
Flow diagram of the sustained attention to response task (SART) experimental design.

We administered SART similar to that used by Christoff et al. (2009), Axelrod et al. (2018) and Kajimura et al. (2019), with periodic thought-probe sampling. The monotonous nature of this task helps to promote high incidences of mind-wandering while allowing to record them both objectively (through reaction times and errors) and subjectively (through thought-probes). We delivered the task using MATLAB 2015b and Psychtoolbox-3 on either a Windows 7 computer (processor: Intel Core i5) or a Macbook Air (macOS High Sierra; 2 GHz Intel Core i7). Each participant used the same computer for all three of their sessions. As seen in Figure 2, we instructed participants to press the space bar as quickly and accurately as possible to the display of a numerical stimulus (0–9), except for the target number 3, where they had to refrain from responding. The target number 3 appeared in 5% of trials in a pseudo-random order (as used in Christoff et al., 2009). Each digit was displayed for 2 s (Christoff et al., 2009) in white Arial size 35 font on a black background in the center of the screen. Between each stimulus, the screen displayed a fixation cross for 20 ms. This is in contrast to Kajimura et al. (2019), where digits were displayed for 1 s followed by 1 s ISI and included ~3.3% targets. Throughout the task, we presented a total of 20 thought probes that asked the participant to reflect on what they were thinking immediately before the probe. While previous studies of tDCS in mind-wandering typically used binary on-task/off-task categories (Axelrod et al., 2015, 2018; Kajimura et al., 2016, 2019), recent research demonstrated that off-task responses to thought-probes during SART are likely to be reflecting both mind-wandering and external distractions (Robison et al., 2019). As we aimed to assess the effects of tDCS on mind-wandering specifically, we instructed participants to provide subjective rates specifically for the extent of task unrelated thoughts that were internally or externally oriented (mind-wandering and external or sensory distractions, respectively).

Each thought-probe appeared at pseudo-random intervals to avoid expectation effects (30, 40, 50, 60, 70, 80 or 90 s; mean = 60 s), similar to Kajimura et al. (2019; ~40–80 s). Participants responded to the probes by a button press on a scale of 1–4 (max to min) using a standard keyboard, with “s,” “e,” “r,” “g” keys for mind-wandering and “h,” “u,” “i,” “l” for external distractions. We instructed them to rate min in both scales on occasions where they were fully on-task. We placed stickers over the key letters to indicate the appropriate number. The task lasted approximately 20 min.

### Design and Procedure

The experiment followed a double-blind, within-subjects design. Participants completed three conditions: anodal stimulation, cathodal stimulation, and sham, in a counterbalanced order and spaced a minimum of 1 week apart (range = 7–23 days, mean = 8.04, SD = 2.62). While previous research typically included anodal and sham conditions only (Kajimura et al., 2019), we were interested in determining whether the reported effects were polarity specific, as this would allow us to establish that any found effects are site-specific (Parkin et al., 2015). At the start of each session, participants completed two practice runs of the SART for a total of 40 min to familiarize themselves with the task and encourage greater levels of mind-wandering as done in Christoff et al. (2009). Participants then completed a baseline run of the SART followed by 20 min of stimulation at rest and concluded with a final post-stimulation run of the SART. It is worth highlighting here that previous research by Kajimura et al. (2019) only included post-stimulation runs of the SART and a very brief practice at the start of the session (not included in their analysis).

For the duration of the full session, participants wore earplugs to reduce external noise distractions. To further reduce auditory and visual distractions, testing was conducted in a quiet room with window blinds closed.

### Statistical Analyses

#### Responses to Thought-Probes

We first categorized the responses to the thought-probes into three categories: “mind-wandering;” “environment;” and “on-task.” For this, we considered a response of 1 or 2 in the 4-point scale to be high and a response of 3 or 4 to be low. We then classified responses of low internal and high external thoughts as “environment,” those of high internal and low external thoughts as “mind-wandering,” and those of low internal and low external thoughts as “on-task.” Occurrences of high (1 or 2) to both internal and external were considered invalid responses and disregarded, this appeared for 18/23 participants and accounted for 8.22% of total responses. Finally, we calculated the frequency/percentage of responses in each of the three categories after these invalid responses were removed.

#### Performance in the SART

We recorded reaction time after the presentation of non-target stimuli and the accuracy of refraining from pressing to target stimuli. Specifically, we defined errors as a button press in response to a target. We interpreted increased commission errors (to targets) and longer reaction times to non-targets as a suggestion of greater levels of mind-wandering.

#### Data Analyses

We used frequentist and equivalent Bayesian comparisons (with default priors) on JASP (JASP Team, 2020). To test the effect of tDCS on our variables of interest (i.e., frequency of mind-wandering, environment, and on-task thoughts, as well as mean reaction time for correct responses to non-targets and number of commission errors on SART), we conducted repeated-measures ANOVAs with stimulation (anodal, cathodal, sham) and session (baseline, post-tDCS) as factors. Finally, to assess the effectiveness of our blinding method, we performed a Chi-Squared test for the association between received and perceived stimulation type (real or sham). We also analyzed the perception of sensations caused by tDCS (intensity, discomfort, tingling, pain, burning, itching) using one-way repeated measures ANOVA.

For the frequentist tests, we set the level of significance at *p* = 0.05. For the Bayesian test, we evaluated both the presence and the absence of an effect by comparing how different models explain the data given the factors of interest (Stafford et al., 2019). We used a Jeffrey-Zellner-Siow Bayes factor (JZS-BF_10_) to contrast the strength of the evidence for models reflecting the null and interactions (Rouder et al., 2012). For ease of interpretation, we include BF_10_ for those >1 and invert them as BF_01_ for those <1. We also calculated a Bayes factor for the exclusion of the variable of interest (BF_excl_) by comparing all models that exclude the interaction with all models that include it. A JSZ-BF between 0.33 and 3 is considered to be weak/anecdotal evidence for an effect; 3–10: substantial evidence; 10–100: strong evidence; >100: very strong evidence (Jeffreys, 1998).

## Results

### Mind-Wandering Responses

**Figure 3:**
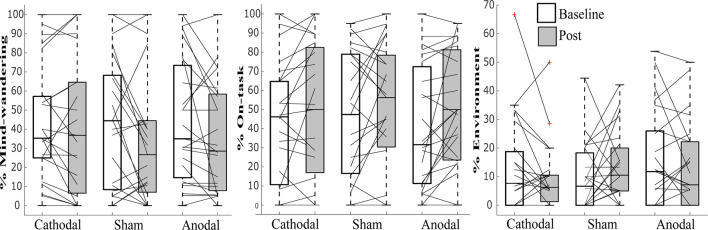
Line graphs with box plots displaying the variability in subjective responses of **(A)** “environment,” **(B)** “on-task,” and **(C)** “mind-wandering” before and after transcranial direct current stimulation (tDCS) in cathodal, sham and anodal conditions. All measures exhibited great variability at baseline and in response to tDCS across all three stimulation conditions.

**Figure 4:**
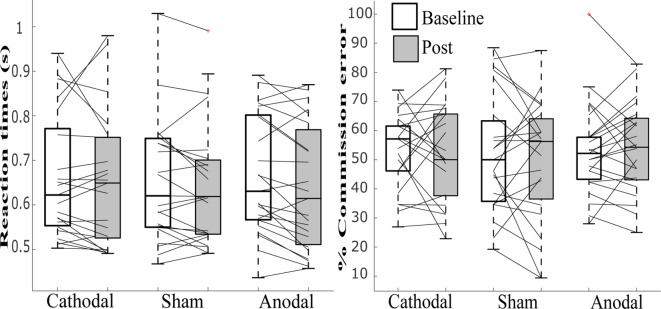
Line graphs with box plots displaying the variability in task performance for **(A)** mean reaction time for the correct response to non-target and **(B)** percent commission errors before and after tDCS in cathodal, sham and anodal conditions. All measures exhibited great variability at baseline and in response to tDCS across all three stimulation conditions.

While the data violated tests for normality, we still employed ANOVA, as this has previously been shown to be robust enough even when assumptions of normality are not met (Schmider et al., 2010). See Table 1 and Figures 3, 4 for summary statistics.

**Table 1:** Percentage of each response type (“mind-wandering,” “on-task,” and “environment”) to thought-probes and performance on sustained attention to response task (SART).

	Baseline			Post-stimulation		
	Sham	Anodal	Cathodal	Sham	Anodal	Cathodal
Mind-wandering (%)	43.14 (33.85)	43.78 (31.89)	43.27 (30.09)	33.82 (33.23)	35.60 (28.88)	41.83 (35.87)
Environment (%)	9.44 (11.54)	15.59 (17.31)	13.34 (9.72)	13.47 (12.47)	14.01 (15.25)	9.73 (11.44)
On-task (%)	47.42 (33.17)	40.62 (32.36)	43.38 (31.58)	52.72 (32.45)	50.40 (30.14)	48.44 (35.29)
Commission errors (%)	51.98 (19.98)	53.21 (15.80)	53.06 (12.70)	49.45 (20.76)	54.06 (15.32)	51.70 (16.46)
Reaction times (s)	0.659 (0.142)	0.668 (0.133)	0.662 (0.137)	0.647 (0.141)	0.638 (0.142)	0.655 (0.150)

*The data are group means (standard deviations in parenthesis)*.

A repeated-measures ANOVA modeled the main effects of stimulation (anodal, cathodal and sham) and session (baseline, post-tDCS), as well as the interaction between them, for the percentage of “mind-wandering,” “environment” and “on-task” responses to probes and commission errors, and mean reaction time (s) to correct responses in the SART. Both frequentist and Bayesian analyses revealed no main effects for responses of “environment,” errors on SART, and no interaction for any of the measures studied. Moreover, Bayesian analyses provided support for the lack of an effect of tDCS (strong to very strong evidence for the null as compared to the full model across all variables, and substantial evidence for the model excluding the interaction, see Table 2).

**Table 2:** Bayesian and frequentists repeated measures ANOVA for the interaction of stimulation (anodal, cathodal, and sham) and session (baseline and post-stimulation) effects on subjective and objective measures of mind-wandering.

	JZS-BF_01_ for the null vs. full	BFexcl (interact)	*F*	*Df*	$\eta_{\text{p}}^{2}$	*P*-value
“Mind-wandering” (%)	43.43	5.19	1.149	2,44	0.050	0.326
“Environment” (%)	115.06	3.67	2.629	2,44	0.107	0.083
“On-task” (%)	13.75	6.40	0.485	2,44	0.022	0.619
Commission errors (%)	244.46	6.79	0.425	2,44	0.019	0.656
Mean reaction time (s)	126.90	5.96	1.005	2,44	0.044	0.374

We found a significant main effect of session in “mind-wandering” propensity in our frequentist ANOVA but inconclusive evidence for this effect in our Bayesian analyses (*F*_(1,22)_ = 4.87; *p* = 0.038, $\eta_{\text{p}}^{2}$ = 0.181, BF_10_ = 1.04), and no significant *post hoc* differences for cathodal (*t*_(22)_ = 0.48, *p* = 0.666, BF_01_ = 4.191) sham (*t*_(22)_ = 1.90, *p* = 0.081, BF_01_ = 1.100) and anodal (*t*_(22)_ = 1.86, *p* = 0.076, BF_01_ = 1.044). We also found a significant main effects of session for “on-task” responses, which was supported by both our frequentist and Bayesian analyses (*F*_(1,22)_ = 6.03; *p* = 0.022, $\eta_{\text{p}}^{2}$ = 0.215, BF_10_ = 3.14), and with a significant increase post-tDCS for anodal (*t*_(22)_ = 2.366, *p* = 0.027, BF_10_ = 2.147) but not cathodal (*t*_(22)_ = 1.382, *p* = 0.181, BF_01_ = 1.980) or sham (*t*_(22)_ = 1.156, *p* = 0.260, BF_01_ = 2.528). For SART performance, we found a main effect of session for RT to correct response to non-targets (*F*_(1,22)_ = 5.741; *p* = 0.026, $\eta_{\text{p}}^{2}$ = 0.207, BF_01_ = 1.74), with significantly faster RTs post-tDCS for anodal (*t*_(22)_ = 2.938, *p* = 0.008, BF_10_ = 6.173) but not cathodal (*t*_(22)_ = 0.470, *p* = 0.643, BF_01_ = 4.136) or sham (*t*_(22)_ = 1.239, *p* = 0.228, BF_01_ = 2.320).

### Post-tDCS Questionnaire

Table 3 displays the proportion of sessions perceived to have had active stimulation, from the post-tDCS perception questionnaire. A Chi-squared test revealed no significant association between participants received and perceived stimulation type (real or sham; $X_{\left(1\right)}^{2}$ = 1.232, *p* = 0.267; BF_01 independent multinomial_ = 1.82, *N* = 69).

**Table 3:** Contingency table displaying the frequency of perceived stimulation type against actual stimulation received.

		Actual	
		Real	Sham
Perceived	Real	34	14
	Sham	12	9

Additional perceptual scales also revealed no significant difference between each type of stimulation as assessed by a one-way repeated measures ANOVA of stimulation (anodal, cathodal, and sham). These include rating of intensity of stimulation (*F*_(2,42)_ = 1.99, *p* = 0.150, *η*^2^ = 0.059; BF_01_ = 1.39); discomfort (*F*_(2,42)_ = 1.57, *p* = 0.221, *η*^2^ = 0.043; BF_01_ = 2.13); tingling (*F*_(2,42)_ = 0.18, *p* = 0.840, *η*^2^ = 0.004; BF_01_ = 7.14); pain (*F*_(2,42)_ = 0.60, *p* = 0.553, *η*^2^ = 0.017; BF_01_ = 4.76); burning (*F*_(2,42)_ = 0.35, *p* = 0.707, *η*^2^ = 0.008; BF_01_ = 5.88); and itching (*F*_(2,44)_ = 0.07, *p* = 0.934; BF_01_ = 7.69). Thus, there was no evidence that participants were able to differentiate between receiving active stimulation or sham (note that BF was inconclusive and therefore there is no conclusive evidence that they were not able either). For all perceptual scores except intensity (where results were inconclusive), there was evidence in support for the physical sensations not differing.

## Discussion

In the present study, we found evidence that tDCS over the right IPL was unable to modulate behavioral incidences of mind-wandering during a SART task. Specifically, we failed to identify a polarity specific effect on either subjective reports of mind-wandering, or errors and reaction times on the task. Our Bayesian analyses provided strong to very strong evidence for the lack of an effect of stimulation in any of our objective and subjective measures of mind-wandering. Specifically, when comparing the null vs. the full models, there was strong evidence for the lack of an effect of tDCS based on incidences of “mind-wandering” and very strong evidence when considering commission errors and reaction times on the task. Furthermore, when looking specifically at the effect of the interaction, we found substantial evidence for the models excluding the interaction as compared to those including it for all three measures, again suggesting no effect of tDCS on objective or subjective reports of mind-wandering. We, therefore, suggest that, against our hypothesis, neither anodal nor cathodal tDCS over the right IPL can influence mind-wandering propensity.

Our findings also contradict earlier reports by Kajimura et al. (2019) showing that stimulation of the right IPL *via* tDCS could modulate mind-wandering propensity during a SART task. This could be due to differences in the operationalization of subjective incidences of mind-wandering. While Kajimura et al. (2019) used a binary “on-task” or “off-task” as possible responses to the thought-probes, we used Likert scales and recorded the level of two types of task unrelated thoughts: mind-wandering and environmental distraction. Previous studies suggest that off-task responses can reflect both mind-wandering and external distraction (Robison et al., 2019) and that both elicit activity on the DMN as compared to on-task instances (Stawarczyk et al., 2011). It is thus possible that our observed lack of effect is due to measuring these two processes independently. This however seems unlikely as, while they both engage the DMN, mind-wandering is associated with greater activity in this network (Stawarczyk et al., 2011). Moreover, most of the distraction observed in our study was due to mind-wandering (with only 9–15% dedicated to external distractions), and our analyses confirmed the lack of an effect for “on task” incidences too. Nevertheless, we cannot rule out that differences in the subject’s experience elicited by the thought probes themselves may have played a role in the discrepancy of findings across both studies. It should be noted that our study was not designed as a direct replication of Kajimura et al. (2019), which alongside other differences discussed above, was conducted in the MRI scanner, and therefore should not be interpreted as such.

Despite the differences in our operationalization of subjective reports of mind-wandering, we were also able to investigate mind-wandering objectively by looking at reaction times and errors on task. According to previous research, the specific methodology used for collecting thought probes (i.e., frequency, framing, or type of responses), or the inclusion of thought probes at all, does not influence the behavioral performance in the task the probes are embedded (Robison et al., 2019; Wiemers and Redick, 2019). Therefore, our reaction times and errors should be comparable to those in previous studies using SART. Similarly to for “mind-wandering” incidences, we found substantial evidence for the lack of an effect of tDCS over performance in the SART (both accuracy and RT). This is in line with previous studies reporting non-significant effects on the accuracy or RTs (Axelrod et al., 2015, 2018; Kajimura et al., 2019).

More importantly, Kajimura et al. (2019) compared mind-wandering reports after tDCS only, between sham and anodal conditions separated by at least a week. It is therefore possible that their results simply reflect subject variability in mind-wandering propensity. Indeed, in our study, we observed large variability in mind-wandering propensity at baseline (ranging from 0% to 100% across participants), which is also consistent with previous reports of mind-wandering outside of the influence of tDCS (Unsworth and McMillan, 2014; Vannucci and Chiorri, 2018). Moreover, Kajimura et al.’s (2019) findings are based on groups of 13 subjects. It is known that small sample sizes can greatly overestimate effect sizes and increase the likelihood of false positives (Lorca-Puls et al., 2018). To overcome these limitations, in the current study, we used a within-subjects design including both baseline and post-tDCS runs on each polarity session with a larger sample size that is almost 2-fold greater (participants: 23). We, therefore, suggest that our design allows for a more robust attribution of the effects (or in this case the lack of) to tDCS. We nevertheless acknowledge that further studies with much larger samples are needed before definite conclusions about the lack of effects of tDCs in mind-wandering can be made. Related to this, it is worth highlighting that, while our Bayesian and Frequentists analyses broadly produced comparable results, there were also some minor inconsistencies (e.g., some of the main effects in our ANOVAs had significant *p*-values but inconclusive BFs). Bayesian statistics are more conservative and known to yield more robust results in studies with relatively small samples (Stegmueller, 2013), and therefore their interpretation should be preferred here. Crucially, as discussed above, both approaches led to consistent results in support of the lack of effect of tDCS in responses to thought probes or performance in the SART.

Possibly other tDCS montages can still successfully modulate mind-wandering. Indeed, some studies have reported modulation of mind-wandering by stimulating the DLPFC (Axelrod et al., 2015, 2018). While the DLPFC is not part of the DMN, it is a central node of the executive network, and therefore an up-regulation of this network may allow higher levels of mind-wandering while maintaining the same level of task performance. Crucially, these findings were not replicated in a recent large-scale study (Boayue et al., 2020). Other studies have suggested that dual stimulation of the DLPFC and right IPL can elicit changes in subjective reports of mind-wandering (Kajimura and Nomura, 2015; Kajimura et al., 2016). However, these results are yet to be replicated outside the original lab. In either case, the disparity of findings may reflect the well-known inter-subject variability in responsiveness to tDCS (López-Alonso et al., 2014; Dyke et al., 2016) and highlight the need to conduct well-powered replications.

A further possible explanation for our lack of effects and the lack of consistency across reports relates to administering tDCS while the participant is at rest. Recent research suggests neuronal activation of the regions targeted for stimulation may be necessary for tDCS to influence the network sufficiently (Kadosh, 2013). For example, studies have shown greater enhancement in performance on a working memory task only when stimulation occurred simultaneously as compared to sham stimulation or tDCS at rest (Andrews et al., 2011). Similarly, tDCS over M1 has a greater influence on motor learning when applied concurrently with the task, as compared to pre-task only (Karok and Witney, 2013). Future studies could, therefore, engage participants in a task known to induce mind-wandering, while administering tDCS, to engage the DMN during stimulation and increase the likelihood of modulating its activity.

Leaving the discussion about the effects of tDCS aside, our analysis of the thought probes across conditions revealed some unexpected findings. Based on prior literature (e.g., McVay and Kane, 2009), we expected to find an increase in mind-wandering in our second run on the SART across conditions (related to having spent more time on the task). In contrast, we failed to show reliable differences for mind-wandering and found contradicting evidence in support of a decrease of on-task responses. We attribute this to the extensive practice run at the start of each session, which would have had a greater effect on our baseline than our post-stimulation runs. Indeed, previous studies using similarly extensive training also failed to identify reliable changes in thought-probe responses with time spent on the task (McVay and Kane, 2009). To further assess this, we conducted additional analyses comparing the first half of each run with the second half. In agreement with the literature, we identified the expected increase in in mind-wandering (*t*_(137)_ = 2.07, *p* = 0.040, BF_01_ = 1.33) and reduction of on-task responses (*t*_(137)_ = 3.33, *p* = 0.001, BF_10_ = 17.75). This suggests our reported main effect of the session was likely explained by the practice session and therefore should not be interpreted as being in contradiction with previous literature.

Overall, we failed to replicate previous research suggesting stimulation of the right IPL alone is capable of modulating mind-wandering propensity and instead provide evidence against an effect, observed through both objective and subjective measures. This, and other recent unsuccessful replications (Boayue et al., 2020), call into question whether it is indeed possible to exert external influence over spontaneous cognitive processes. Future studies should include brain imaging to further understand what influence tDCS is exerting on the target networks and elucidate whether the reported inconsistency in behavioral effects is indeed reflecting unsuccessful neural modulations or subthreshold changes in brain activity.

## Data Availability Statement

Datasets are available on request.

## Ethics Statement

The studies involving human participants were reviewed and approved by the University of Birmingham’s Science, Technology, Engineering and Mathematics Ethical Review Committee. The patients/participants provided their written informed consent to participate in this study.

## Author Contributions

SC and DF-E designed the research. SC supervised data collection and analyzed the data. SC and DF-E interpreted the data and wrote the manuscript. All authors contributed to the editing of the manuscript.

## Conflict of Interest

The authors declare that the research was conducted in the absence of any commercial or financial relationships that could be construed as a potential conflict of interest.

## Footnotes

^1^www.simnibs.org
